# Mechanistic Evaluation of Antimicrobial Lipid Interactions with Tethered Lipid Bilayers by Electrochemical Impedance Spectroscopy

**DOI:** 10.3390/s22103712

**Published:** 2022-05-13

**Authors:** Sue Woon Tan, Won-Yong Jeon, Bo Kyeong Yoon, Joshua A. Jackman

**Affiliations:** 1 School of Chemical Engineering and Translational Nanobioscience Research Center, Sungkyunkwan University, Suwon 16419, Korea; suewoon4695@gmail.com (S.W.T.); powerwy@skku.edu (W.-Y.J.); 2 School of Healthcare and Biomedical Engineering, Chonnam National University, Yeosu 59626, Korea

**Keywords:** electrochemical impedance spectroscopy, tethered bilayer lipid membrane, antimicrobial lipid, surfactant, membrane disruption

## Abstract

There is extensive interest in developing real-time biosensing strategies to characterize the membrane-disruptive properties of antimicrobial lipids and surfactants. Currently used biosensing strategies mainly focus on tracking membrane morphological changes such as budding and tubule formation, while there is an outstanding need to develop a label-free biosensing strategy to directly evaluate the molecular-level mechanistic details by which antimicrobial lipids and surfactants disrupt lipid membranes. Herein, using electrochemical impedance spectroscopy (EIS), we conducted label-free biosensing measurements to track the real-time interactions between three representative compounds—glycerol monolaurate (GML), lauric acid (LA), and sodium dodecyl sulfate (SDS)—and a tethered bilayer lipid membrane (tBLM) platform. The EIS measurements verified that all three compounds are mainly active above their respective critical micelle concentration (CMC) values, while also revealing that GML induces irreversible membrane damage whereas the membrane-disruptive effects of LA are largely reversible. In addition, SDS micelles caused membrane solubilization, while SDS monomers still caused membrane defect formation, shedding light on how antimicrobial lipids and surfactants can be active in, not only micellar form, but also as monomers in some cases. These findings expand our mechanistic knowledge of how antimicrobial lipids and surfactants disrupt lipid membranes and demonstrate the analytical merits of utilizing the EIS sensing approach to comparatively evaluate membrane-disruptive antimicrobial compounds.

## 1. Introduction

There is broad interest in utilizing biosensing technologies to help address global health challenges, especially in the context of developing improved diagnostic and therapeutic measures [1]. One of the most promising application areas involves drug development, in which case biosensors can offer mechanistic viewpoints on relevant interaction processes that complement and expand on biological testing [2,3]. Such capabilities have emerged as a promising approach to address the issue of antibiotic-resistant bacteria, which are a major threat to public health due to the overuse and misuse of antibiotics [4]. The slow development of new antibiotics and the accelerating spread of antimicrobial resistance have highlighted the urgent need to develop alternatives to antibiotics [5,6,7]. To address this issue, the use of naturally occurring antimicrobial lipids as antibiotic alternatives has been extensively studied [8,9]. Indeed, antimicrobial lipids composed of single-chain lipid amphiphiles along with structurally related surfactants have been explored as membrane-disruptive compounds that can damage bacterial cell membranes [10,11,12]. Microbiological testing has further revealed that antimicrobial lipids, such as fatty acids and monoglycerides can inhibit bacteria by killing bacterial cells and/or reducing cell viability [13,14]. Additional studies have shown that fatty acids and monoglycerides can also inhibit membrane-enveloped viruses [15,16], supporting that these compounds might not only replace antibiotics, but also demonstrate a broader set of antimicrobial functions. As such, there is growing interest to understand how antimicrobial lipids and surfactants disrupt lipid membranes from a mechanistic perspective.

To date, the post-treatment effects of antimicrobial lipids have often been investigated on bacterial cell membranes through cell-based permeability, viability, or growth assays or microscopy imaging techniques such as scanning electron microscopy and transmission electron microscopy [10,17,18]. To gain insight into the corresponding mem-brane-disruptive interactions and associated mechanisms of action that can guide com-pound selection, potency determination, and/or formulation development, there have also been ongoing efforts to utilize real-time biosensing techniques to track how antimicrobial lipids disrupt model lipid membranes that mimic the basic lipid bilayer architecture of bacterial cell membranes and are compatible with surface-sensitive measurement techniques. These efforts fit within the broader landscape of developing label-free biosensors and biomedical sensors that scrutinize biomacromolecular interactions for use in new directions such as pharmaceutical drug development, which complement the traditional focus on molecular detection [19,20].

For example, the supported lipid bilayer (SLB) platform has been employed with acoustic and optical biosensing techniques to study how antimicrobial lipids and surfactants cause changes in membrane morphology, which can be distinguished in a com-pound-specific manner and are correlated with the critical micelle concentration (CMC) of each compound [10,21,22,23]. Indeed, such approaches have worked well for characterizing strain-induced morphological changes, such as membrane budding or tubulation, which result from membrane interactions, while it should be noted that these morphological changes are related to the confined SLB geometry and occur in order to relieve strain. At the same time, there is also interest in investigating how antimicrobial lipid interactions affect membrane permeability, which is directly related to the interaction process itself and also associated with antimicrobial activity [17,18,24]. In this regard, the electrochemical impedance spectroscopy (EIS) technique is a potentially useful technique to track membrane permeability changes in a label-free manner, especially since it has been previously shown to detect peptide-induced membrane disruption with high sensitivity compared to other conventional biosensing options [25].

While SLB platforms are useful model membrane platforms, the lipid bilayer is only separated from the underlying solid support (typically silica) by an ultrathin interfacial water layer and hence the ionic reservoir is quite small, which has led to the development of the tethered bilayer lipid membrane (tBLM) platform with a larger ionic reservoir and other suitable features for EIS measurements. The tBLM platform involves the covalent attachment of long, tether-like anchor lipids onto a gold surface with relatively low surface coverage, followed by forming the tethered lipid bilayer by vesicle fusion or solvent-exchange type processes [26,27]. The anchor lipid composition consists of a mixture of two distinct molecules—the anchors themselves as well as spacers—that compete for binding to the gold surface during a solution-phase incubation step, resulting in a mixed monolayer of long anchors and short spacers that can be varied depending on their relative molar concentrations in the bulk solution [28]. The anchors are amphiphilic molecules that provide a template to guide tBLM self-assembly, whereas the short spacers have no direct contact with the lipid membrane and function to passivate the gold surface and create the ionic reservoir. Hence, the choice of anchor lipid and spacer influences membrane packing density and integrity, which consequently affect the electrical sealing properties of the tBLM [26]. For example, increasing the length of the tethers can lead to increased fluidity (e.g., permeability) of the resulting membrane, formation of undulated membranes, pore-like defects, and even tether detachment from the surface [29]. With appropriate tBLM platform design and electrical sealing, the relatively large ionic reservoir between the lower bilayer leaflet and gold surface makes it possible to measure electrical current flow through the membrane [30,31]. Given these capabilities, EIS measurements have been conducted to study the mechanism of action of membrane-disruptive antimicrobial peptides using the tBLM platform, which has proven useful for distinguishing peptides that induce transverse pore formation [32], membrane lysis [33,34], and size modulation of existing membrane pores [35,36]. Hence, there is excellent potential to apply the EIS sensing technique together with the tBLM platform to characterize the membrane-disruptive interactions of antimicrobial lipids and surfactants from a mechanistic perspective.

Towards this goal, herein, we conducted EIS measurements to investigate the mem-brane-disruptive properties of representative antimicrobial lipids and surfactants, namely glycerol monolaurate (GML), lauric acid (LA), and sodium dodecyl sulfate (SDS), using the tBLM platform. GML and LA are two of the most biologically active monoglycerides and fatty acids, respectively, while SDS is a widely used surfactant that has a similar chain structure. Our main objective was to evaluate the concentration-dependent measurement responses for each compound in terms of corresponding CMC values, and to establish an analytical basis for gaining insight into how the different compounds disrupt lipid membranes. While antimicrobial peptides have well-developed models to describe their mechanisms of actions, such perspectives are less well developed for antimicrobial lipids and surfactants and the EIS measurement capabilities utilized here help to address this gap while establishing a broadly applicable biosensing tool to mechanistically profile antimicrobial lipids and surfactants that are relevant to healthcare, biotechnology, and food science applications.

## 2. Materials and Methods

### 2.1. Materials

Glycerol monolaurate (GML), lauric acid (LA), and sodium dodecyl sulfate (SDS) were purchased from Sigma Aldrich (St. Louis, MO, USA). Phosphate-buffered saline (PBS, pH 7.4) was obtained from Gibco (Carlsbad, CA, USA). All solutions were prepared in Milli-Q-treated deionized water (MilliporeSigma, Burlington, MA, USA).

### 2.2. Antimicrobial Lipid Preparation

Stock solutions of GML and LA were prepared by dissolving the appropriate amount of compound in ethanol to 200 mM stock concentration. The aliquots were then diluted in aqueous buffer to the highest test concentration of 500 µM and 2 mM for GML and LA, respectively, before experiments. The SDS sample was prepared by weighing the desired amount, followed by dissolving the sample in aqueous buffer to the highest test concentration of 2 mM. To enhance solubility, each sample was heated at 70 °C for 30 min prior to preparing two-fold serial dilutions. The diluted samples were cooled down to room temperature before adding them to the measurement chamber.

### 2.3. Tethered Bilayer Lipid Membrane (tBLM) Formation

The tBLM was fabricated on top of a pre-tethered benzyl-disulfide ethylene glycol T10 monolayer that consisted of 90% spacer (hydroxyl terminated benzyldisulphide tetra-ethylene glycol) and 10% tether (benzyldisulphide polyethylene glycol phytanyl) molecules attached to the gold electrode slide (product code: SDx-BG), as supplied by SDx Tethered Membranes (Sydney, Australia). The gold electrode slide was placed over a tethaPlate cartridge (product code: SDx-T10, SDx Tethered Membranes) according to the standard configuration (see also Figure S1). Based on this configuration, the solvent ex-change technique was performed to form the complete tBLM, as previously described [32]. First, an 8 µL aliquot of a 3 mM mobile lipid phase that consisted of 70% zwitterionic C20 diphytanyl-diether-phosphatidylcholine lipid and 30% C20 glycerol diphytanyl ether (DPEPC) lipid dissolved in ethanol (product code: SDx-S1, SDx Tethered Membranes), was added on top of the tethered monolayer in each of the six chambers within a single tethaPlate cartridge (SDx Tethered Membranes). After a 2 min incubation period, each chamber was rinsed thrice with 100 µL PBS each time. The tBLM formation process was characterized by electrochemical impedance spectroscopy (EIS) measurements using the tethaPod instrument (product code: SDx-R1, SDx Tethered Membranes). All EIS measurements were performed in alternating current (AC) mode, and swept-frequency impedance spectroscopy with a 25 mV AC excitation (see also refs. [28,37,38]) and frequency range of 0.1 Hz to 2000 Hz was used. No offset voltage was applied. For Bode plots, note that only the frequency range up to 1000 Hz is presented in the graphs because relevant signal changes occurred within that range. Conductance (G_m_) and capacitance (C_m_) values of around ~0.3 µS and 1.2–1.6 µF/cm^2^ respectively, for the 2.1 mm^2^ electrode were considered to be acceptable baseline values [32]. Data collection and processing were conducted by using the tethaQUICK (product code: SDx-B1, SDx Tethered Membranes) and OriginPro (OriginLab, Northampton, MA, USA) software packages. All measurements were repeated at least twice and representative measurements are shown. The deviation in measurement values for the tBLM platform was less than 5% between experiments. Note that the G_m_ and C_m_ signals were recorded every 3 min and each data point is represented by a circular symbol while lines connect the dots as a guide for the eyes.

## 3. Results

### 3.1. Measurement Strategy

**Figure 1:**
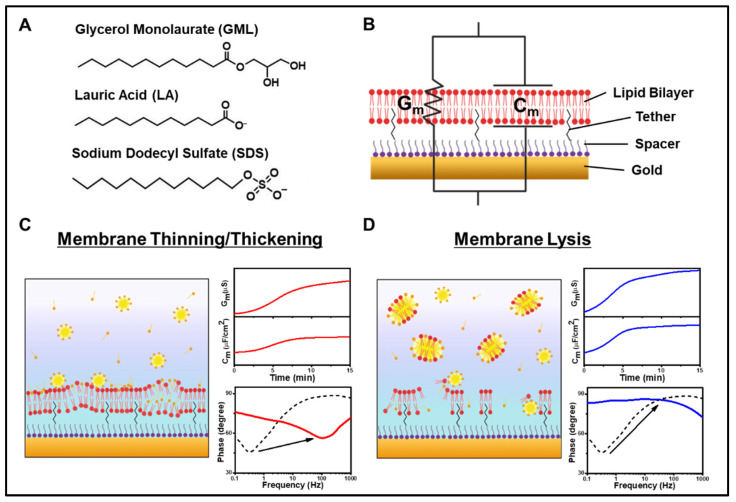
(**A**) Molecular structures of GML, LA, and SDS. (**B**) Schematic illustration of EIS measurement setup with the tBLM platform. The corresponding electrical properties of the tethered lipid bilayer are modelled in terms of conductance (G_m_) and capacitance (C_m_). (**C**,**D**) Schematic illustration of (**C**) membrane thinning/thickening and (**D**) membrane lysis effects and typical measurement responses.

The glycerol monolaurate (GML) monoglyceride and lauric acid (LA) fatty acid were selected as representative antimicrobial lipids along with the structurally related sodium dodecyl sulfate (SDS) surfactant to evaluate their corresponding membrane-disruptive behaviors using the tBLM platform. As presented in Figure 1A, GML and LA each have a saturated, 12-carbon long hydrocarbon chain while their headgroups are different. In the GML case, the headgroup is a glycerol group while the headgroup is a carboxylic acid group in the LA case. At pH 7.4, GML is nonionic whereas LA is negatively charged because its carboxylic acid headgroup is deprotonated [11]. Similarly, SDS is an anionic detergent that consists of a saturated, 12-carbon long hydrocarbon chain with a negatively charged sulfate headgroup. Although all three molecules have similar hydrocarbon chains, their distinct headgroups contribute to different physiochemical properties such as net charge and critical micelle concentration (CMC) values. Of note, previous findings have reported that the three compounds are principally active at bulk concentrations above their respective CMC values and can induce distinct membrane morphological changes in supported lipid bilayers accordingly [24]. Such insights provided guidance to select appropriate compound concentrations for testing in the present tBLM experiments.

To investigate the membrane-disruptive effects of these compounds, electrochemical impedance spectroscopy (EIS) measurements were conducted using tethered bilayer lipid membrane (tBLM) platforms. After establishing the measurement baseline signals, GML, LA, or SDS was added to the tBLM platform and the incubation period lasted for 30 min, followed by a PBS buffer washing step. A schematic illustration of the tBLM platform with the EIS measurement setup is shown in Figure 1B. Prior to experiment, a monolayer of anchor lipids was covalently attached to the gold electrode surface via gold-sulfur dative bonding and the anchor lipid mixture consisted of anchor lipids possessing a tether unit that binds the lipid to the solid support and an exposed hydrophobic lipid part that acts as the bottom leaflet of the tethered lipid bilayer, along with a spacer moiety that separates the tether units laterally [39]. The monolayer was formed by incubating an ethanolic solution of dissolved tether and spacer molecules in a 9:1 molar ratio with the gold electrode surface, followed by ethanol rinsing [38]. Afterwards, the complete lipid bilayer architecture of the tBLM platform was formed by incubating the monolayer-functionalized gold electrode surface with an ethanolic solution of dissolved 70% zwitterionic C20 diphytanyl-diether-phosphatidylcholine lipid and 30% C20 glycerol diphytanyl ether (DPEPC) lipid. A buffer rinsing step was performed in a solvent-exchange type process [40] in order to induce a phase transition of the lipid molecules whereby they self-assemble with one another along with the attached tethered lipids to form a full-spanning tBLM across the gold electrode surface, which includes an ionic reservoir between the gold surface and lower lipid leaflet.

The tBLM platform was integrated with the EIS measurement setup to temporally track the conductance (G_m_) and capacitance (C_m_) values of the tBLM platform in response to compound addition and resulting membrane-disruptive effects. G_m_ is related to the passage of ionic current through the lipid bilayer, while C_m_ measures the electrical charge buildup in the lipid bilayer. As an initial control experiment, blank PBS was added in lieu of compound in PBS and no changes in the G_m_ or C_m_ signals occurred, thus verifying that EIS measurement responses arise from compound addition and not due to buffer exchange/washing alone (Figure S2). In addition to these two signals, we also analyzed the Bode plot of phase versus log_10_ [frequency]. Operationally, EIS measures the impedance across the tBLM by applying an alternating current over a range of frequencies. The phase difference between the voltage and current is represented as the phase angle and indicates the tendency of the tBLM platform to act as a capacitor [41]. The variation in phase angle with respect to frequency is displayed as the Bode plot. Briefly, the frequency at the phase minima reflects G_m_ and the phase value at the phase minima reflects C_m_. Conceptually, when ion leakage across the tethered lipid bilayer occurs, there will be a larger G_m_ signal that causes the phase minima to shift to a higher frequency. Alternatively, when membrane thinning occurs, there will be a large C_m_ signal that causes the phase minima to shift to a higher phase value [42].

Based on these measurement capabilities, we proceeded to evaluate the membrane-disruptive effects of the different compounds. In general, two commonly observed types of membrane disruption are (1) membrane thinning/thickening and (2) membrane lysis. In the first case, the test compound can intercalate within the tethered lipid bilayer [43], resulting in changes in membrane thickness and fluidity (Figure 1C). Such changes in turn affect membrane permeability as well; increased permeability is typically associated with G_m_ and C_m_ shift increases along with a prominent increase in frequency and/or modest increase in phase minima in the phase profile [33,34]. On the other hand, in the second case of membrane lysis, the tethered lipid bilayer surface tension is reduced, followed by membrane solubilization whereby lipid molecules leave the tBLM surface and results in membrane defects (Figure 1D). Membrane lysis typically induces large increases in the G_m_ and C_m_ values along with a frequency increase and a very large increase in the phase minima in the phase profile [33].

### 3.2. GML

**Figure 2:**
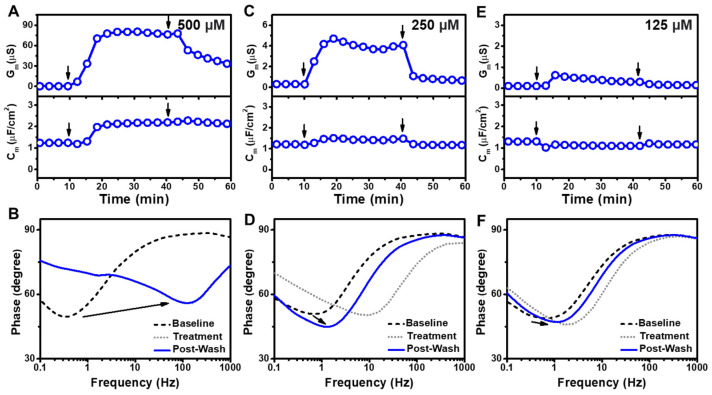
(**A**) Time-resolved conductance (G_m_) and capacitance (C_m_) shifts upon 500 µM GML addition to the tBLM platform. The arrows indicate compound addition at *t* = 10 min and buffer washing at *t* = 40 min, respectively. (**B**) Bode plots for 500 µM GML addition to the tBLM platform, whereby baseline, treatment, and post-washing reflect the initial measurement signal, signal after compound addition, and signal after buffer washing, respectively. Corresponding data for (**C**,**D**) 250 µM GML and (**E**,**F**) 125 µM GML addition to the tBLM platform.

To investigate the membrane-disruptive properties of GML, we treated the tBLM platform with different GML concentrations above and below its CMC value of 60 μM (ref. [11]) and measured the corresponding measurement responses (Figure 2). At the highest test concentration, 500 µM GML rapidly induced G_m_ and C_m_ shift increases of around 80 µS and 0.93 µF/cm^2^, respectively (Figure 2A). The measurement responses plateaued at those values and, after buffer washing, both G_m_ and C_m_ shifts decreased to around 30 µS and 0.85 µF/cm^2^, respectively, relative to the initial tBLM baseline. The corresponding Bode plots indicated a shift to higher frequencies and a modest increase in the phase minima, which indicates irreversible membrane defect formation (Figure 2B). Of note, the Bode plots after compound treatment and after subsequent buffer washing in this case were essentially identical and superimposed graphically, indicating that 500 µM GML treatment caused irreversible membrane-disruptive effects.

Similar interaction kinetics were observed in the case of 250 µM GML treatment, albeit the recorded G_m_ and C_m_ shift increases were around 20-fold smaller at 4.42 µS and 0.32 µF/cm^2^, respectively (Figure 2C). Following a subsequent rinsing step, the final G_m_ and C_m_ shifts were around 0.43 µS and 0.02 µF/cm^2^, respectively, relative to the baseline values. From the Bode plots, it was observed that the phase minima shifted to higher frequencies upon GML addition (Figure 2D). Upon rinsing, the phase minima shifted to lower frequencies and a smaller phase, suggesting that the membrane defects caused by GML were partially reversible. This response profile could be explained by the partial insertion of the hydrophobic part of GML into the hydrophobic region of the membrane [33,44,45], and the partially inserted GML was then removed from the membrane with the subsequent rinsing step, leaving some defects in the membrane.

On the other hand, treatment with 125 µM GML led to even smaller G_m_ and C_m_ shifts of around 0.52 µS and −0.15 µF/cm^2^, respectively, and the corresponding values after buffer washing were around ~0 µS and −0.12 µF/cm^2^, respectively (Figure 2E). The Bode plots showed only a slight increase in the frequency range upon 125 µM GML addition while the signal was reversed back to near the baseline after buffer washing (Figure 2F). These responses indicate that the GML caused some membrane defects, but the membrane returned to nearly its original state after washing. Compared to the higher GML concentration cases, these results support that lower GML concentrations caused less extensive, irreversible membrane disruption. Furthermore, at even lower GML concentrations around the CMC and below, the G_m_ and C_m_ shifts were nearly negligible, indicating that GML had only minor effect in those cases (Figure S3). Collectively, the data support that GML is mainly active above its CMC and exhibits greater membrane-disruptive effects at higher GML concentrations within that range.

### 3.3. LA

**Figure 3:**
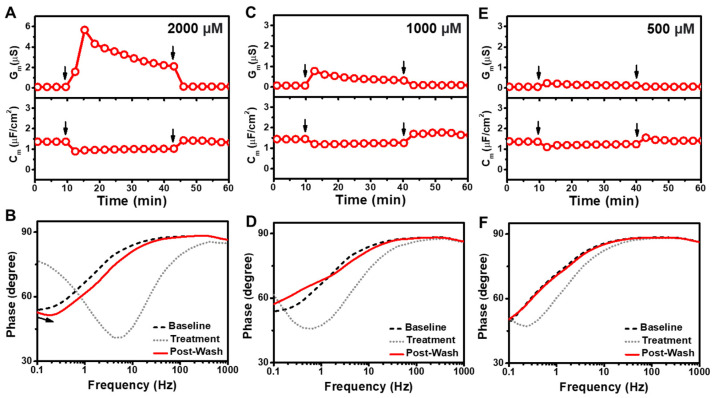
(**A**) Time-resolved conductance (G_m_) and capacitance (C_m_) shifts upon 2000 µM LA addition to the tBLM platform. The arrows indicate compound addition at *t* = 10 min and buffer washing at *t* = 40 min, respectively. (**B**) Bode plots for 2000 µM LA addition to the tBLM platform, whereby baseline, treatment, and post-washing reflect the initial measurement signal, signal after compound addition, and signal after buffer washing, respectively. Corresponding data for (**C**,**D**) 1000 µM LA and (**E**,**F**) 500 µM LA addition to the tBLM platform.

We proceeded to characterize the membrane-disruptive properties of LA and treated the tBLM platform with different LA concentrations above and below its CMC value of 950 μM (ref. [11]) (Figure 3). The addition of 2000 µM LA induced a transient G_m_ shift increase of 5.6 µS along with a C_m_ shift decrease of −0.41 µF/cm^2^ (Figure 3A). After buffer washing, the final G_m_ and C_m_ values nearly returned to baseline values with shifts of only around 0.05 µS and −0.05 µF/cm^2^, respectively, relative to the initial signals. The corresponding Bode plots showed a frequency increase and decrease in the phase minima due to LA addition, while the phase profile nearly recovered to baseline levels after buffer washing (Figure 3B). Similarly, the addition of 1000 µM LA caused a transient G_m_ shift increase of 0.71 µS along with a C_m_ shift decrease of −0.19 µF/cm^2^, which indicates only minor membrane-disruptive effects (Figure 3C). After buffer washing, the final G_m_ and C_m_ values were 0.02 µS and 0.19 µF/cm^2^, respectively, relative to the initial signals. The largely reversible effects of 1000 µM LA treatment were also reflected in the Bode plot (Figure 3D).

Upon 500 µM LA treatment, there were only minor G_m_ and C_m_ shifts of 0.17 µS and −0.26 µF/cm^2^, respectively, which is consistent with the presence of LA monomers only at that concentration (Figure 3E). After buffer washing, the final measurement shifts were negligible, with G_m_ and C_m_ shift values of ~0 µS and 0.04 µF/cm^2^, respectively, and the corresponding Bode plot showed no change relative to the baseline (Figure 3F). These results are consistent with LA only exhibiting membrane-disruptive effects above the CMC and lower LA concentrations further below the CMC were also inactive (Figure S4). Together, these findings support that LA micelles exhibit membrane-disruptive effects of the tBLM platform during the interaction process, although such effects are largely reversible.

### 3.4. SDS

**Figure 4:**
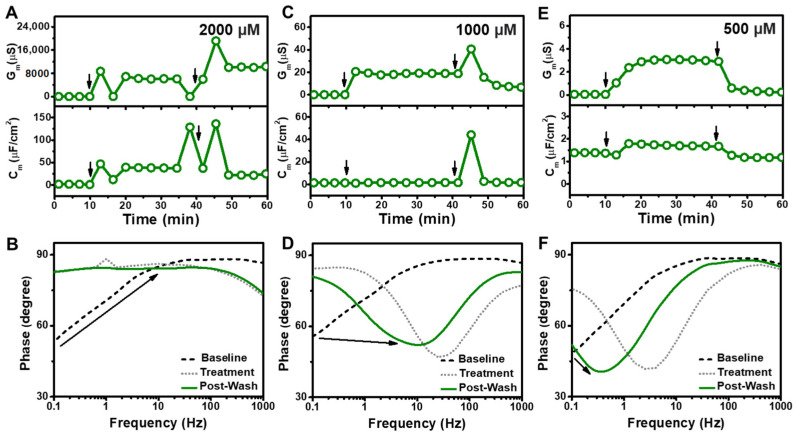
(**A**) Time-resolved conductance (G_m_) and capacitance (C_m_) shifts upon 2000 µM SDS addition to the tBLM platform. The arrows indicate compound addition at *t* = 10 min and buffer washing at *t* = 40 min, respectively. (**B**) Bode plots for 2000 µM SDS addition to the tBLM platform, whereby baseline, treatment, and post-washing reflect the initial measurement signal, signal after compound addition, and signal after buffer washing, respectively. Corresponding data for (**C**,**D**) 1000 µM SDS and (**E**,**F**) 500 µM SDS addition to the tBLM platform.

In addition to GML and LA, we investigated the membrane-disruptive properties of SDS and treated the tBLM platform with different SDS concentrations above and below its CMC value of 800 μM (ref. [11]) (Figure 4). At the highest tested concentration, 2000 μM SDS addition caused rapid and large G_m_ and C_m_ shifts of up to 8700 µS and 45 µF/cm^2^, respectively (Figure 4A). In marked contrast to the other tested compounds, the measurement responses showed extensive fluctuations indicating loss of membrane integrity and buffer washing caused the G_m_ and C_m_ shifts to further increase to around 19,000 µS and 134 µF/cm^2^, respectively, before stabilizing at around 10,000 µS and 31 µF/cm^2^. The corresponding Bode plots indicated a very large increase in the phase minima that is consistent with membrane lysis (Figure 4B) [33]. Similarly, 1000 μM SDS addition induced a rapid G_m_ shift of 20.5 µS, whereas the initial C_m_ shift was negligible and around −0.31 µF/cm^2^ (Figure 4C). Upon buffer washing, both the G_m_ and C_m_ shifts initially increased up to around 40.5 µS and 42.8 µF/cm^2^, respectively, before stabilizing at 6.39 µS and 0.30 µF/cm^2^. In this case, the Bode plots indicated a frequency increase that is consistent with membrane defect formation (Figure 4D). By contrast, 500 μM SDS addition to the tBLM platform caused relatively minor G_m_ and C_m_ shifts that stabilized at values around 2.93 µS and 0.35 µF/cm^2^, respectively (Figure 4E). In that case, buffer washing led to G_m_ and C_m_ shift decreases of around 0.18 µS and −0.18 µF/cm^2^, respectively, which were distinct from the transient increases observed in the higher SDS concentration cases described above. The Bode plots further showed a decrease in the phase minima along with a frequency increase that is indicative of membrane defect formation (Figure 4F).

**Figure 5:**
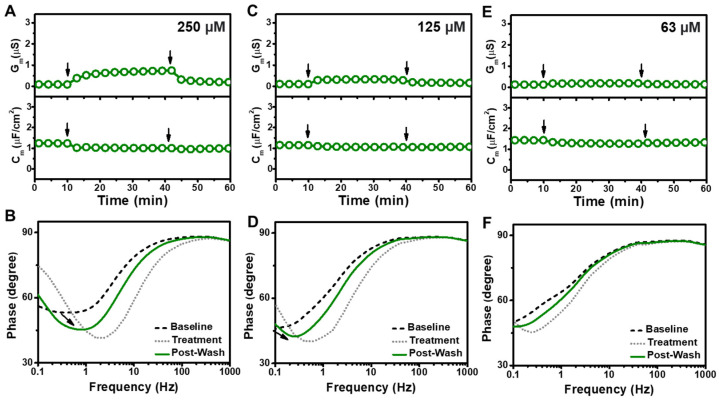
(**A**) Time-resolved conductance (G_m_) and capacitance (C_m_) shifts upon 250 µM SDS addition to the tBLM platform. The arrows indicate compound addition at *t* = 10 min and buffer washing at *t* = 40 min, respectively. (**B**) Bode plots for 250 µM SDS addition to the tBLM platform, whereby baseline, treatment, and post-washing reflect the initial measurement signal, signal after compound addition, and signal after buffer washing, respectively. Corresponding data for (**C**,**D**) 125 µM SDS and (**E**,**F**) 63 µM SDS addition to the tBLM platform.

On the other hand, 250 µM SDS addition caused more subtle membrane-disruptive effects with gradual G_m_ and C_m_ shifts of around 0.7 µS and 0.21 µF/cm^2^, respectively (Figure 5A). The corresponding Bode plots showed a decrease in the phase minima along with a modest frequency increase, which indicated membrane defect formation (Figure 5B). Likewise, 125 µM SDS addition caused more subtle membrane-disruptive effects with gradual G_m_ and C_m_ shifts of around 0.2 µS and −0.05 µF/cm^2^, respectively, along with similar but smaller changes in the Bode plots that point to a relatively minor extent of membrane defect formation (Figure 5C,D). There were negligible changes in the measurement signals when 63 µM SDS was added, with G_m_ and C_m_ shifts of only 0.05 µS and −0.11 µF/cm^2^, respectively, and only minor changes in the Bode plots (Figure 5E,F). Together, these data support that extensive, solubilization-like disruption of the tBLM platform mainly occurred when the SDS concentration was above its CMC and showed concentration-dependent behavior, while SDS exhibited more modest membrane-disruptive effects at SDS concentrations as low as 125 µM.

## 4. Discussion

**Figure 6:**
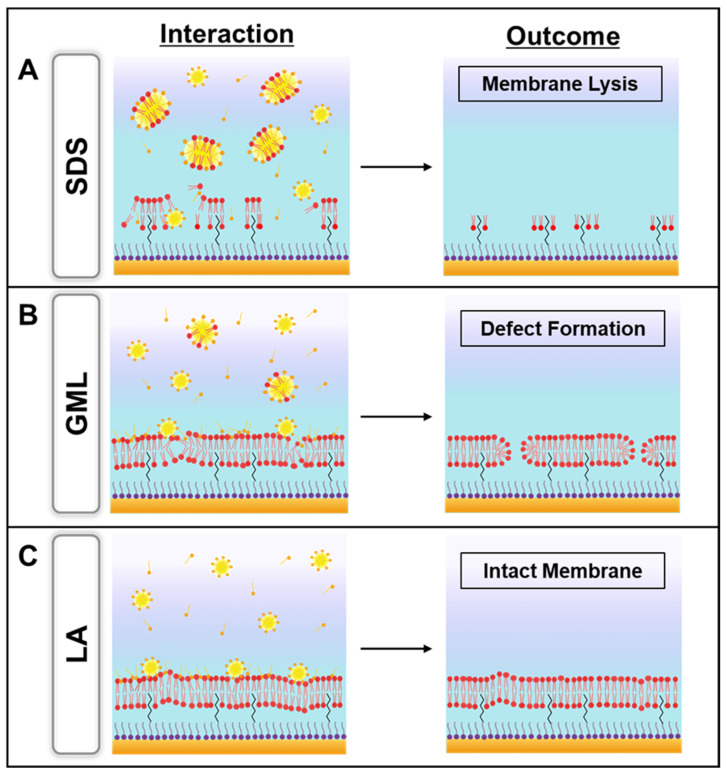
Schematic summary illustrating the membrane-disruptive effects of (**A**) SDS, (**B**) GML, and (**C**) LA micelles on tethered bilayer lipid membrane platforms. The inferred scenarios reflect the EIS measurement trends observed in each case and are proposed based on the wealth of antimicrobial peptide data that has been obtained for tBLM platform experiments using the EIS technique in past reports [33,36].

In this study, we performed EIS experiments to gain mechanistic insight into how GML, LA, and SDS affect the electrochemical properties of the tBLM platform. Compared to past measurement approaches that were mainly sensitive to changes in three-dimensional membrane morphology, the EIS technique enabled real-time, label-free characterization of changes in lipid bilayer permeability and electrical sealing properties. Based on the EIS results, Figure 6 presents a schematic summary to explain the membrane-disruptive behaviors of the three compounds and each case is described one-by-one below. The membrane-disruptive effects of all three compounds primarily occurred above their respective CMC values, and hence the following discussion pertains to when each compound was in the micellar state.

We begin by describing the SDS case because it had the most pronounced membrane-disruptive behavior and its solubilizing activity caused membrane lysis (Figure 6A). SDS has a relatively large headgroup and its membrane insertion caused lipid removal from the tBLM platform due to SDS-lipid complex formation and micellization [46]. Consequently, the loss of membrane integrity disrupted the electrical sealing of the tBLM platform, which resulted in large increases in the conductance and capacitance signals [33]. By contrast, GML did not cause extensive membrane solubilization but instead caused irreversible membrane defect formation (Figure 6B). The ability to cause membrane defect formation is likely related to the nonionic character of GML, whereby it can readily translocate across the two bilayer leaflets. Even so, notably, the membrane integrity in this case was still largely preserved, as indicated by relatively stable sealing properties after buffer washing.

On the other hand, LA exhibited a distinct pattern of membrane-disruptive behavior because the corresponding effects were largely reversible and the tBLM platform retained membrane integrity (Figure 6C). This finding was supported by the phase profiles in the Bode plots and can be rationalized by taking into account that LA has an anionic headgroup, which limits its membrane translocation ability [10]. Accordingly, LA intercalation affected membrane packing in the tBLM platform during the interaction stage but the weakly interacting LA molecules were readily rinsed away during the buffer washing step [47]. As such, all three compounds demonstrated unique membrane-disruptive properties, especially in the cases of GML and LA that induced irreversible and reversible membrane damage, respectively. While past studies using other biosensing techniques have shown that GML is generally more potent than LA [48], our findings obtained using the EIS technique in the present study newly establish that GML is not only more potent (active at lower bulk concentrations) but also causes irreparable membrane damage in a distinct manner to that of LA.

Such findings demonstrate the utility of label-free biosensing strategies to study complex biomacromolecular interaction processes, whereas conventional biological assays focus on post-treatment effects rather than the interaction process itself [18,49]. Previously used label-free biosensing techniques such as the quartz crystal microbalance-dissipation (QCM-D) and localized surface plasmon resonance (LSPR) sensing revealed that GML, LA, and SDS induce membrane budding, tubule formation, and membrane solubilization, respectively, above their corresponding CMC values and established that CMC is a key factor that influences the difference in antimicrobial potency of the various compounds [10,22]. Indeed, the QCM-D and LSPR measurement techniques are sensitive to the architectural configuration of the supported lipid bilayer platform and can detect compound-triggered changes in three-dimensional membrane morphology that arise from the confined membrane geometry and accompanying strain relief in applicable cases, while it would be desirable to utilize a label-free biosensing approach that can more sensitively detect changes in membrane permeability due to compound interactions with a less confined, more biomimetic membrane platform.

Towards this goal, in this study, we explored using the tBLM platform because it is less confined on the surface compared to the supported lipid bilayer platform due to sparse tethering, which enables more dynamic and biologically relevant changes in membrane properties. Moreover, the tBLM platform is compatible with the EIS measurement technique, which is highly sensitive to detect changes in membrane permeability that are associated with membrane-disruptive antimicrobial activities. Hence, the EIS measurements were able to distinguish mechanistic differences in how GML, LA, and SDS disrupt lipid membranes based on the real-time conductance and capacitance signals along with the phase profile. Importantly, the distinct membrane-disruptive properties of GML and LA against lipid membranes contribute to understanding why GML possesses greater antimicrobial activity than LA. In line with these past biophysical and biological results, our findings support that GML has greater membrane-disruptive potency than LA and SDS on account of a lower CMC value while unraveling distinct types of membrane interactions for each compound, including how SDS micelles and monomer can both cause membrane disruption to different extents. From this viewpoint, the EIS measurements provide new capabilities for studying the membrane-disruptive properties of antimicrobial lipids and surfactants.

In terms of analogizing the current experimental results to antimicrobial peptide cases that have previously been studied using the tBLM platform, the EIS data further support that the membrane-disruptive behaviors of GML and LA bear resemblance to the carpet model. Specifically, the permeabilizing effect of these compounds is likely related to the parallel orientation of inserted compound molecules in the tethered lipid bilayer, resulting in a ‘carpet’ that perturbs membrane packing [33,50,51]. In the GML case, this carpet-like behavior coupled with high translocation ability resulted in irreversible membrane defect formation, whereas LA also exhibited carpet-like behavior but only had low translocation ability, which resulted in reversible membrane interactions instead.

## 5. Conclusions

In this work, we investigated the membrane-disruptive effects of three representative antimicrobial lipids and surfactants on the tBLM platform by utilizing the EIS measurement technique. A key benefit of this sensing approach was that changes in membrane electrochemical properties could be directly evaluated to determine how the test compounds affect the ionic permeability of the lipid membranes, which represents a distinct vantage point to past studies that mainly looked at resulting effects on three-dimensional membrane morphology. Moreover, the tBLM platform has a higher degree of structural flexibility (i.e., less confined geometry) compared to previously used confined SLB platforms, which allowed us to distinguish the irreversible and reversible membrane damaging effects caused by GML and LA micelles, respectively. Moreover, SDS was observed to cause distinct types of membrane-disruptive behavior above and below its CMC, which is also in line with recent findings that the membrane-disruptive properties of SDS depend on the membrane nanoarchitecture [52]. Together, these findings demonstrate that the EIS technique is useful for evaluating the mechanistic details of how antimicrobial lipids and surfactants interact with the tBLM platform and such capabilities can be utilized to further screen and optimize the membrane-disruptive performance of candidate lipids and surfactants in future work.

## Data Availability

The raw data required to reproduce these findings are available from the corresponding authors on reasonable request.

## Supplementary Materials

The following supporting information can be downloaded at: https://www.mdpi.com/article/10.3390/s22103712/s1, Figure S1: Experimental setup for EIS measurements. Figure S2: Time-resolved conductance (G_m_) and capacitance (C_m_) shifts and Bode plots for negative control experiment; Figure S3: Time-resolved conductance (G_m_) and capacitance (C_m_) shifts and Bode plots upon 63 µM, 31 µM, and 16 µM GML addition to the tBLM platform; Figure S4: Time-resolved conductance (G_m_) and capacitance (C_m_) shifts and Bode plots upon 250 µM LA addition to the tBLM platform.
