# Supplementary material for: Mechanistic Evaluation of Antimicrobial Lipid Interactions with Tethered Lipid Bilayers by Electrochemical Impedance Spectroscopy
Source: Sensors (Basel). 2022 May 13;22(10):3712. doi: 10.3390/s22103712 (PMC9148023; doi:10.3390/s22103712)
Supplement: Supplementary file 1 [file sensors-22-03712-s001.zip › sensors-1707818-supplementary.pdf]

sensors

MDPI

Supplementary Information

Mechanistic Evaluation of Antimicrobial Lipid Interactions with Tethered Lipid Bilayers by Electrochemical Impedance Spectroscopy

Sue Woon Tan [1], Won-Yong Jeon [1], Bo Kyeong Yoon [2,*] and Joshua A. Jackman [1,*]

[1] School of Chemical Engineering and Translational Nanobioscience Research Center, Sungkyunkwan University, Suwon 16419, Korea; suewoon4695@gmail.com (S.W.T.); powerwy@skku.edu (W.-Y.J.)
[2] School of Healthcare and Biomedical Engineering, Chonnam National University, Yeosu 59626, Korea
* Correspondence: bkyoon@jnu.ac.kr (B.K.Y.); jjackman@skku.edu (J.A.J.)

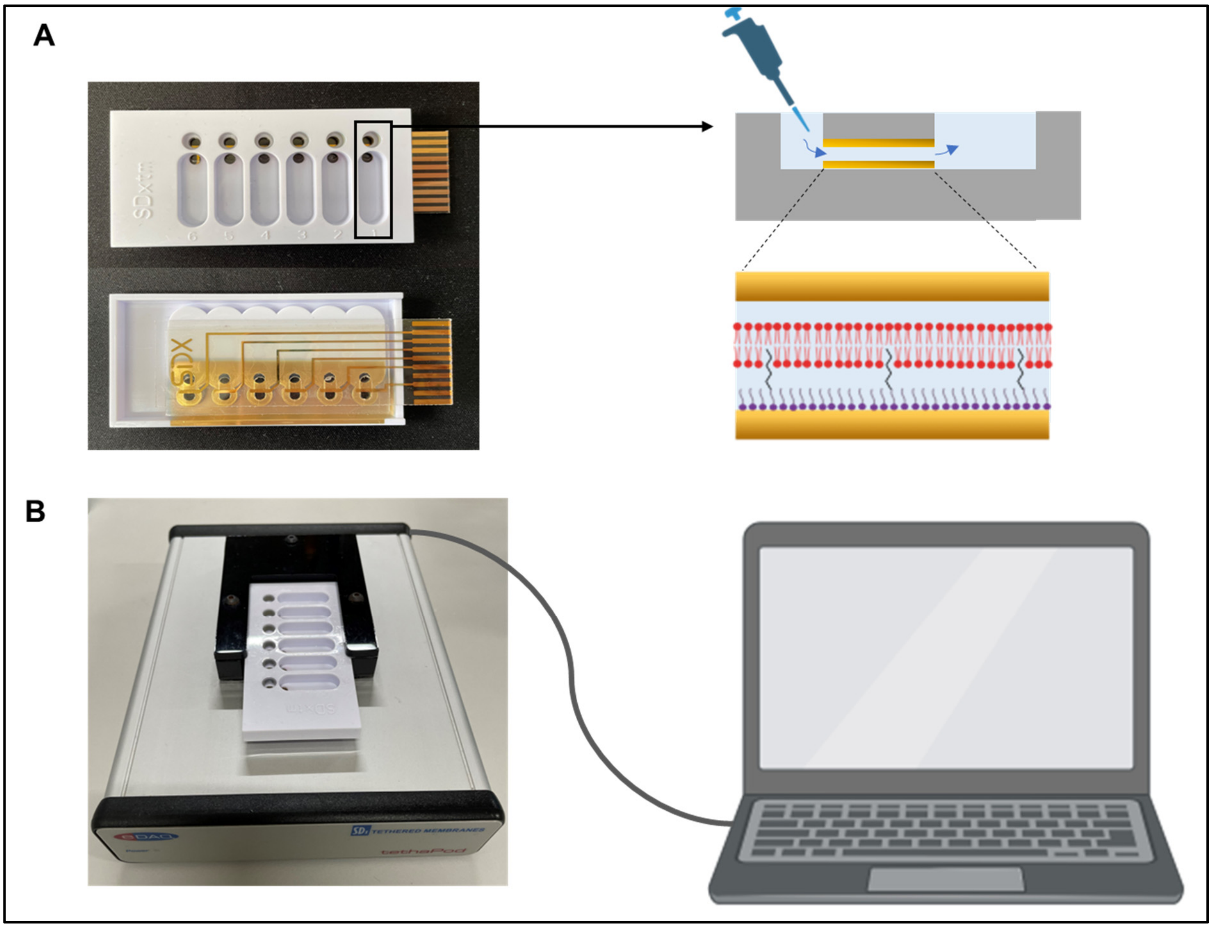

Figure S1. Experimental setup for EIS measurements. (**A**) Assembly of patterned gold electrode slide with flow cell cartridge. One cartridge contains six chambers. Cross section of each chamber shows the presence of the gold electrode surface to enable tBLM formation *via* the solvent-exchange method. (**B**) Flow cell cartridge containing the tBLM platforms was attached to the tethaPod reader, which was connected to a computer *via* USB port for obtaining signal outputs.

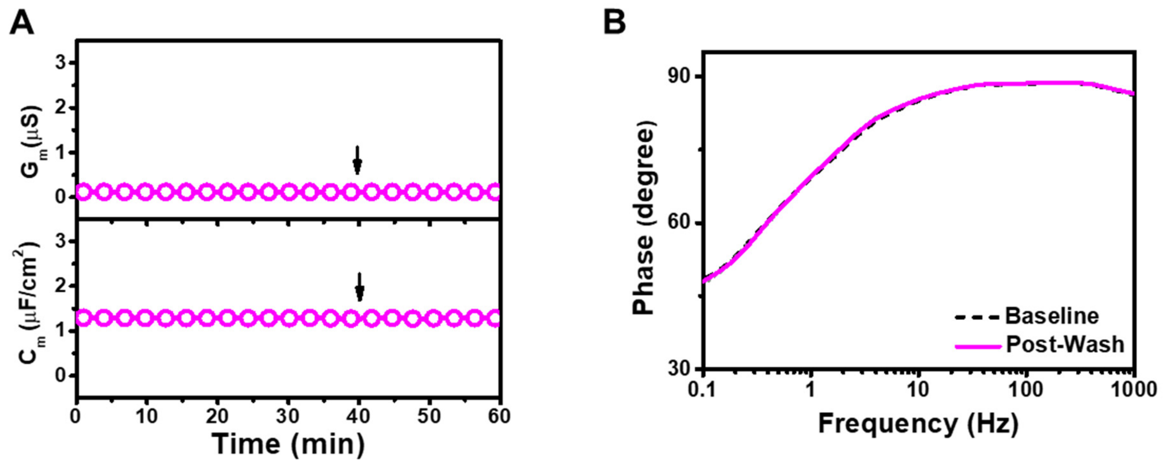

Figure S2. (**A**) Time-resolved conductance (G_m) and capacitance (C_m) shifts of negative control experiment. The arrow indicates buffer washing at t = 40 min. (**B**) Bode plots for negative control experiment, whereby baseline and post-washing reflect the initial measurement signal and signal after buffer washing, respectively.

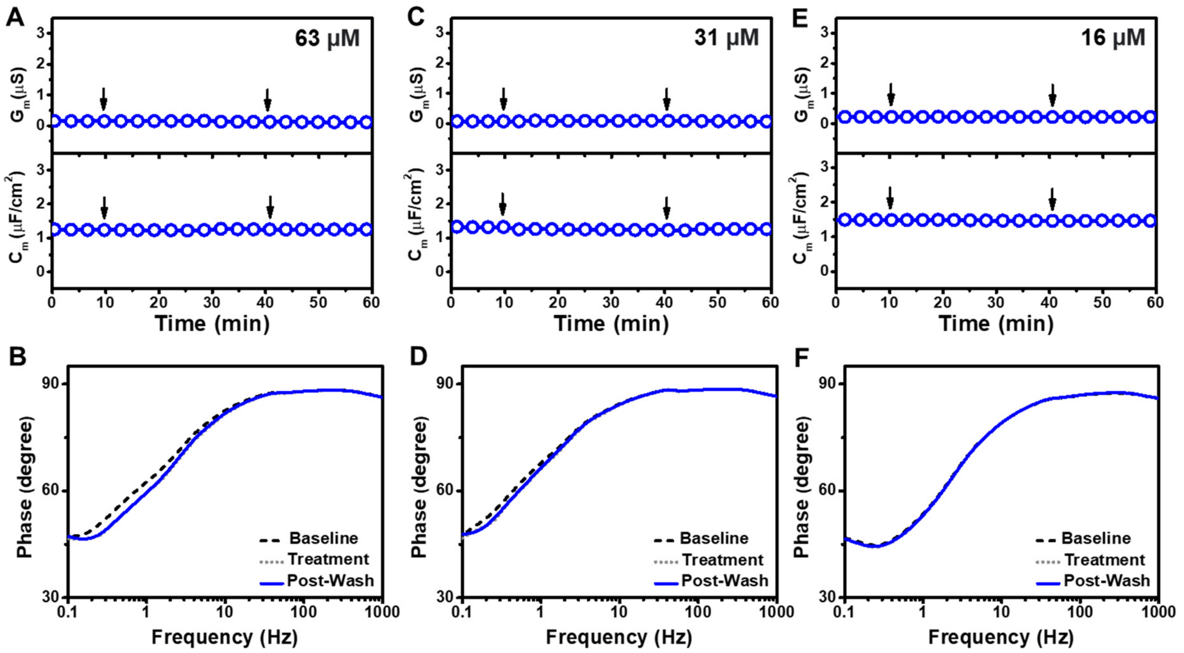

Figure S3. (**A**) Time-resolved conductance (G_m) and capacitance (C_m) shifts upon 63 μM GML addition to the tBLM platform. The arrows indicate compound addition at t = 10 min and buffer washing at t = 40 min, respectively. (**B**) Bode plots for 63 μM GML addition to the tBLM platform, whereby baseline, treatment, and post-washing reflect the initial measurement signal, signal after compound addition, and signal after buffer washing, respectively. Corresponding data for (**C**,**D**) 31 μM GML and (**E**,**F**) 16 μM GML addition to the tBLM platform.

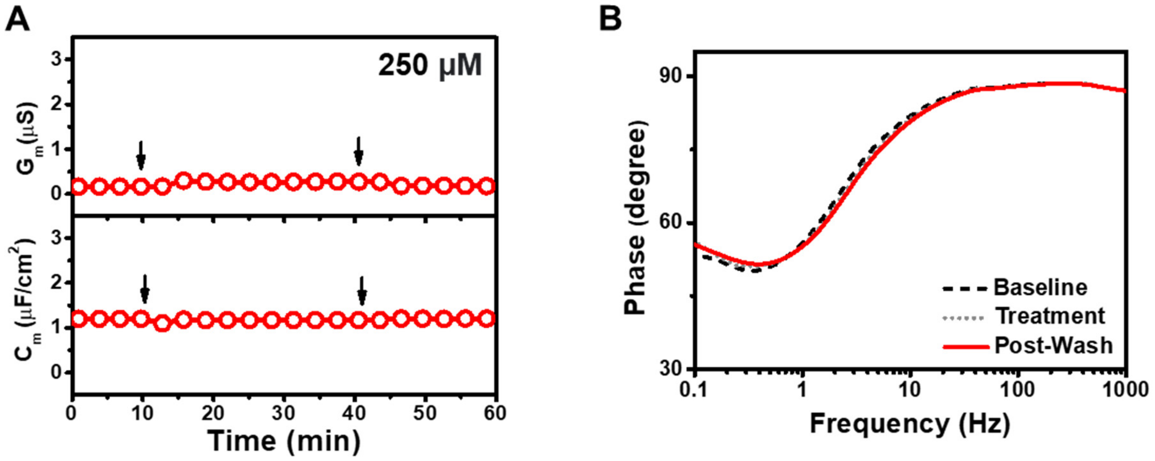

Figure S4. (**A**) Time-resolved conductance (G_m) and capacitance (C_m) shifts upon 250 μM LA addition to the tBLM platform. The arrows indicate compound addition at t = 10 min and buffer washing at t = 40 min, respectively. (**B**) Bode plots for 250 μM LA addition to the tBLM platform, whereby baseline, treatment, and post-washing reflect the initial measurement signal, signal after compound addition, and signal after buffer washing, respectively.
